# Ultrafast optomechanical pulse picking

**DOI:** 10.1007/s00340-016-6608-4

**Published:** 2017-01-16

**Authors:** Nikolai Lilienfein, Simon Holzberger, Ioachim Pupeza

**Affiliations:** 1 Max-Planck-Institut fuer Quantenoptik, Hans-Kopfermann-Strasse 1, 85748 Garching, Germany; 2 Present Address: Menlo Systems GmbH, Am Klopferspitz 19a, 82152 Martinsried, Germany

**Keywords:** Pulse Train, Output Coupler, Deflection Angle, Astigmatism, Beam Path

## Abstract

State-of-the-art optical switches for coupling pulses into and/or out of resonators are based on either the electro-optic or the acousto-optic effect in transmissive elements. In high-power applications, the damage threshold and other nonlinear and thermal effects in these elements impede further improvements in pulse energy, duration, and average power. We propose a new optomechanical switching concept which is based solely on reflective elements and is suitable for switching times down to the ten-nanosecond range. To this end, an isolated section of a beam path is moved in a system comprising mirrors rotating at a high angular velocity and stationary imaging mirrors, without affecting the propagation of the beam thereafter. We discuss three variants of the concept and exemplify practical parameters for its application in regenerative amplifiers and stack-and-dump enhancement cavities. We find that optomechanical pulse picking has the potential to achieve switching rates of up to a few tens of kilohertz while supporting pulse energies of up to several joules.

## Introduction

Picking individual pulses from the MHz-repetition-rate pulse trains produced by (amplified) laser oscillators is particularly important in the context of high-pulse-energy lasers. Here, pulse pickers are necessary to reduce the repetition rate of the seed oscillator pulse train before amplification in average-power-limited laser systems. Other prominent applications are the direct extraction of pulses from laser resonators (cavity dumping) [1], and the coupling of pulses into and out of the cavities of regenerative amplifiers [2–4]. Cavity dumping has also been demonstrated in passive external resonators known as enhancement cavities (ECs) [5, 6]. While this concept has not yet found widespread application, it has recently encountered renewed interest in the context of high-pulse-energy Yb-based amplifiers [7, 8]. The relevant properties of pulse pickers are the switching time and switching rate, efficiency, contrast, and optical bandwidth. Equally important for intracavity applications are properties which affect the transmitted pulses, i.e., losses, chromatic dispersion, and, for high-power applications, self-induced nonlinear and thermal effects.

Cavity dumping from oscillators and regenerative amplifier cavities typically relies on Pockels cells. These devices use the electro-optic Pockels effect in nonlinear crystals to rapidly rotate the polarization of the intracavity pulse upon application of a high voltage. Depending on its polarization, the pulse can be subsequently coupled out by means of a polarizer. The length of the crystals necessary to reach a polarization rotation sufficient for efficient switching is typically tens of millimeters. Pockels cells include several antireflection-coated surfaces which create losses. Since the electro-optic effect scales linearly with the electric field, the half-wave voltage increases with the aperture width. This, together with the availability of large crystals of sufficient quality, makes increasing the aperture of the cell technologically challenging [3]. The damage threshold of crystals and antireflective coatings, as well as nonlinear and thermal effects, are the limiting factors for the extractable peak and average power from regenerative amplifiers [3, 9]. State-of-the-art systems achieve pulse energies of 30 mJ at 10 kHz repetition rate and 200 mJ at 1 kHz [10]. For amplification, the pulses are typically stretched to durations in the nanosecond range [4, 11].

In contrast to the cavities of laser oscillators and of regenerative amplifiers, which include gain media, the increase in pulse energy in ECs depends on the correct temporal and spatial overlap of circulating and seeding pulses, and the growth per round-trip is typically small. Thus, losses, dispersion, and other distortions of the intracavity pulse are critical, and rule out the use of Pockels cells for efficient dumping. For proof-of-principle demonstrations of the stack-and-dump concept, acousto-optic modulators (AOMs) have been employed [5, 12, 13]. Here, the beam is coupled out by a transient grating, induced by an acoustic wave propagating in a solid, e.g., a fused silica plate. The switching time of these devices is determined by the beam size and by the speed of sound in the material. A sufficiently short switching time to pick pulses from a megahertz pulse train requires the beam to be tightly focused in the AOM. Recently, the extraction of 0.16 mJ pulses from an EC has been demonstrated [14]. The pulses were chirped to a duration of 2.5 ns, and the output repetition rate was 30 kHz. In particular, the high nonlinearity caused by the small beam size in the AOM represents a bottleneck for the further scaling of such systems [14].

Recently, a mechanical pulse picker based on a concept similar to a chopper wheel has been proposed as a possible way to circumvent these limitations [7]. Here, a moving mirror that periodically intercepts the circulating pulse would act as the switching element, avoiding the losses, dispersion, nonlinearities, and thermal lensing associated with intracavity transmissive elements. However, the centrifugal force that would occur in a chopper wheel spinning fast enough to couple out single pulses even from a tightly focused MHz-repetition-rate pulse train would be close to the limits given by the tensile strength of potential rotor materials. This poses a major technological challenge and would severely limit the capabilities of the such devices. Even if feasible, this output coupler would represent a high-precision element of considerable size, complexity, and cost.

In this article, we present a novel concept for an optomechanical pulse picker consisting of exclusively reflective optics. It uses rotating mirrors and stationary imaging mirrors in order to move an entire section of a beam path without affecting the beam path outside of this section. While sharing the advantages of the chopper wheel, the concept drastically reduces the mechanical demands on the rotor by using the beam path as a lever. We present three particular geometries and discuss their advantages and drawbacks. We find that devices based on this concept could be suitable to pick joule-level pulses from optical cavities with repetition rates of several tens of megahertz, at rates of several kilohertz.

## Circular single-mirror geometry

**Fig. 1:**
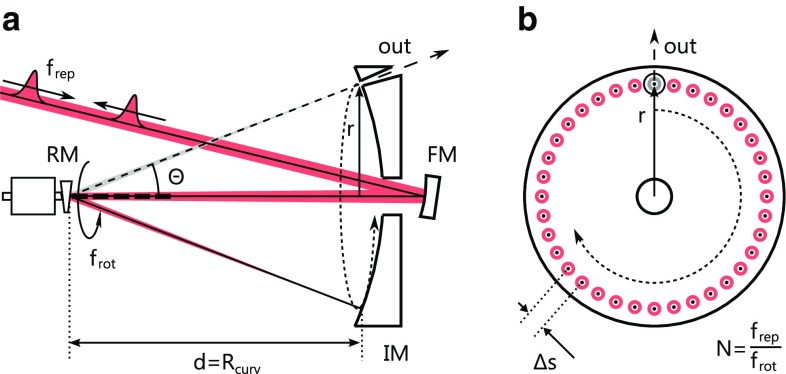
**a** Side view of the circular single-mirror geometry comprising one rotating mirror (RM), a spherical imaging mirror (IM, radius of curvature $R_\text {curv}$) with an opening, and a curved folding mirror (FM). The beam path is shown for two rotation angles (*dashed line* beam path for output coupling). For a full rotation, the deflected beam path describes a cone. **b** Front view of the imaging mirror with *N* points of incidence of individual pulses from the incoming pulse train being distributed along a circle of radius *r*

Figure 1a shows the most basic variant of the concept. It is based on a mirror that rotates at a high angular velocity. The normal of its flat surface is tilted by a small angle from its rotation axis, such that the rotating mirror reflects a stationary input beam into a time-dependent deflection beam path. The rotating mirror surface is positioned in the center of curvature of a large spherical mirror. The deflection beam path impinges orthogonally on the stationary mirror surface for all rotation angles, and is reflected back along its incident path. Irrespective of the deflection angle, the second pass on the rotating mirror deflects the output beam along a path coinciding with the input path. Since the stationary spherical mirror images the surface of the rotating mirror onto itself, we refer to it as the imaging mirror. To achieve collimated input and output beams, a curved folding mirror can be used to focus the beam on the imaging mirror. Figure 1b illustrates the points where individual pulses from a pulse train impinge on the imaging mirror. If the deflection paths of subsequent pulses are separated by a distance $\Delta s$ that is larger than the beam diameter, mirrors in the deflection beam path or openings in the imaging mirror can be used to pick pulses from the pulse train.

The switching time $\tau$ of the pulse picker is given by the velocity with which the beam moves along the circle of radius *r*, and the 1/$e^2$ beam radius *w* in that plane. If we require that $\Delta s = 2 w$, we find that

1 $$ \tau = \frac{ w }{\pi r f_{\text {rot}}}, $$

with $f_\text {rot}$ being the revolution rate of the rotating mirror. This equation also holds for a chopper-wheel-based pulse picker. In this case, the value of *r*, is given by the size of the chopper wheel. For our optomechanical pulse picker, on the other hand, it depends on the deflection angle from the rotating mirror $\varTheta$ and on the distance to the imaging mirror *d*, according to $r=d \sin (\varTheta )$. The beam path is used as a lever, and the diameter of the rotor needs to be just large enough to provide a sufficient aperture for the transverse beam size. The switching rate is equal to the revolution rate. A magnetic bearing of the rotor is desirable to achieve a high rotation speed as well as a high stability of the rotor and of its revolution rate, a long lifetime, and to allow for operation in vacuum. In [15], a suitable self-bearing motor reaching a speed of 8.4 kHz intended for laser scanning applications has been demonstrated. With a length of 55 mm and a diameter of about 30 mm, the motor is quite compact. With this motor, and, e.g., an imaging mirror of 15 cm diameter and a beam radius of 50 μm in the focal plane, the switching time is 25.3 ns, corresponding to a maximum pulse repetition rate of close to 40 MHz. Precise holes with diameters in the range of several tens of μm can be manufactured, e.g., via laser drilling [16]. To allow for reliable pulse picking, the points of incidence of the pulses on the imaging mirror have to be fixed. To this end, the repetition rate of the pulse train has to be locked to an integer multiple of the rotation frequency. In addition, the pulse train has to be stabilized to a specific fixed phase with respect to the rotation phase. Both of these requirements are achievable with standard technologies.

A pulse picker of this kind could be used in a linear resonator cavity, with the imaging mirror being one of the end mirrors. The number of round-trips during one rotation period is $N=f_\text {rep}/f_\text {rot}$, with $f_\text {rep}$ being the repetition rate of the resonator. The transverse separation of the paths of successive pulses is given by

2 $$\begin{aligned} \Delta s_1 = 2 \pi d \sin (\varTheta )\frac{f_{\text {rot}}}{f_{\text {rep}}} . \end{aligned}$$

When the input beam path coincides with the rotation axis of the rotating mirror, the angle of incidence on its surface is constant, and equals half the deflection angle $\varTheta$. The polarization of the beam with respect to the imaging mirror surface turns with the rotating mirror. The spectral phase and reflectivity of dielectric mirror coatings typically show increasing polarization dependence with a larger angle of incidence and with an increasing spectral bandwidth. Even though the imaging setup does not affect the polarization geometrically, this effect will cause a birefringent spectral phase modulation increasing with the angle of incidence and the required bandwidth. Any rotation-angle-dependent modulation is periodic with $f_\text {rot}$, leading to a constant output of the system at $f_\text {rot}$. For some applications , however, small angles of incidence and, thus, a long distance *d* may be necessary.

For large values of *d*, the propagation time $\Delta t = 2d/c$ between the first and the second pass of the pulse on the rotating mirrors becomes relevant. During this time, the mirror rotates by $\Delta \phi = 2 \pi f_\text {rot} \Delta t$. This rotation results in an effective tilt $\beta$ between the vectors normal to the mirror surface for the first and second passes given by

3 $$\begin{aligned} \sin \Big (\frac{\beta }{2}\Big )=\sin \Big (\frac{\varTheta }{2}\Big )\sin \Big (\frac{\Delta \phi }{2}\Big ). \end{aligned}$$

For the example mentioned above, a distance $d={0.5}\hbox {m}$ corresponding to $\varTheta ={8.6}^{\circ }$, results in an effective tilt of 13.5 μrad.

A limitation of this type of geometry for high-power applications is presented by the high intensity on the imaging mirror. The beam radius *w* on the imaging mirror depends on the focusing power of the curved folding mirror and on the caustic of the surrounding resonator. A small beam waist reduces the necessary separation of adjacent spots, but increases the peak and average intensity on the imaging mirror. While no transmissive elements prone to thermal and nonlinear effects are used, mirror damage will occur for high peak intensities or fluences. Additionally, thermal lensing in mirrors can affect the operation of cavities at high average powers [17, 18]. While the overall size of the pulse picker can be scaled up with the required peak and average power, the necessity of focussing the beam on a cavity mirror is generally disadvantageous for high-power applications.

## Circular double-mirror geometry

**Fig. 2:**
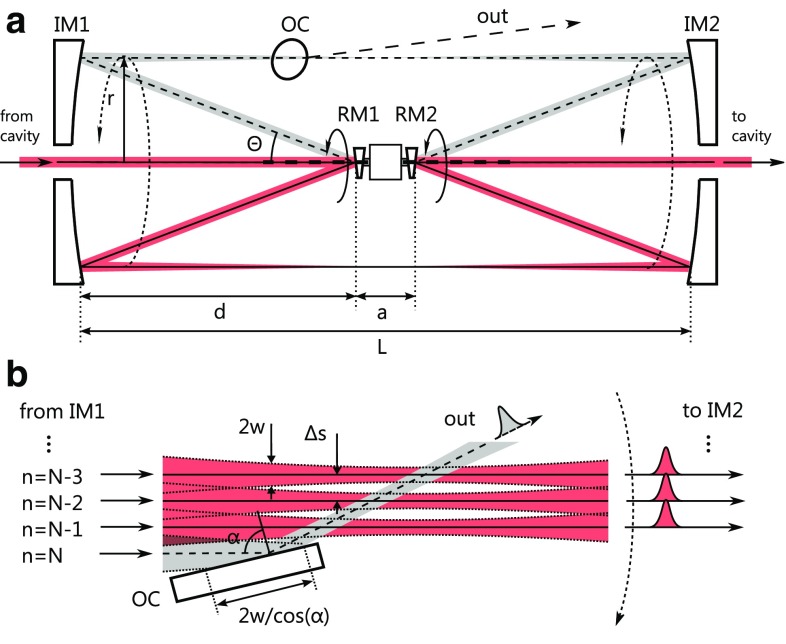
**a** Side view of the circular two-mirror geometry with two rotating mirrors (RM1, RM2), two imaging mirrors (IM1, IM2) and an output coupling mirror (OC). The beam path is shown for two rotation angles (*dashed line* beam path just before output coupling). For a full rotation, the deflected beam path describes a cylinder between the imaging mirrors. **b** Schematic view of the grazing-incidence output coupling mirror. The beam path is shown for the three final round-trips of the pulse before the output coupling event (*dashed line*)

Figure 2 shows a second variant of the concept which mitigates the above problem. Here, two rotating mirrors and two imaging mirrors are used in a setup which is symmetric with respect to a central plane. The rotating mirrors are fixed on a single shaft which is driven by the motor. The curved imaging mirrors are arranged such that their focal points coincide with the center of the rotating mirror surfaces, analogously to a 4-f imaging configuration. The first rotating mirror reflects the input beam into a deflection beam path that depends on its rotation angle. The two imaging mirrors guide the deflected beam to the second rotating mirror, which produces a stationary output beam. Between the rotating mirrors, some space is needed for the bearing and power unit of the rotor, separating their surfaces by a distance *a*. When the distance between the imaging mirrors *L* is large with respect to *a* and the deflection angle $\varTheta$ is small, the imaging mirror configuration is close to a true 4-f imaging. In this case, the deflection beam is focused in the symmetry plane of the setup when the input and output beams are nearly collimated. For larger values of *a*, the beam has to be slightly divergent at the entrance of the pulse-picker setup and slightly convergent at the exit. A mirror placed at some point along the deflected beam path between the imaging mirrors can be used for input or output coupling of the pulse (Fig. 2b). For some applications, the resonator cavity may remain blocked for a small fraction of the rotation period after output coupling and/or before input coupling. Then, the picking mirrors can be larger than the beam size at the position of input/output coupling. The space just before or behind the coupling mirrors can also be used for the mechanical support of the rotor unit. The surface of the stationary mirrors can be either spherical or parabolic. Spherical mirrors are simpler to align and manufacture, but exhibit astigmatism when hit at nonzero angles of incidence. Note that the sagittal and tangential planes with respect to the rotating and imaging mirrors, and thus the astigmatism, are rotating together with the beam path. Particularly in resonators operated close to an edge of the stability range [19], this astigmatism will result in a rotating ellipticity of the cavity mode. Similar to the birefringent effects in dielectric mirror coatings mentioned before, this can be mitigated by decreasing the angle of incidence at the cost of increased size.

An optomechanical pulse picker of the second variant could be used in both linear and ring resonator cavities. For small angles of incidence on the mirrors, the following equation gives the pulse path separation for the second variant:

4 $$\begin{aligned} \Delta s_2 = \pi ( L - a ) \tan (\varTheta )\frac{f_{\text {rot}}}{f_{\text {rep}}} . \end{aligned}$$

It is similar to the one for the single-mirror geometry, but the total length, given here by the distance of the imaging mirrors *L*, doubles for otherwise identical parameters. In this geometry, the rotation of the mirrors during the propagation time along the deflection beam path can be compensated by adjusting the orientation of the rotating mirrors with respect to each other. In contrast to the single-mirror geometry, the spot size of the beam is large on both the rotating and the imaging mirrors. On the pulse-picking mirrors (output or input couplers), the beam size can be chosen corresponding to the required switching time by changing the position along the deflection beam path (Fig. 2b).

While the average power impinging on the picking mirrors is far lower than on the other optics, the small spot size necessary to achieve switching times below 100 ns causes a considerably higher peak intensity. The intensity on the output coupler can be reduced by placing it under grazing incidence. For instance, at an extreme angle of incidence $\alpha$ of ${89}^{\circ }$, the irradiated area would be increased by a factor of about 57. Generally, the peak fluence F of a beam with a Gaussian profile on the output coupler is

5 $$\begin{aligned} F = \frac{2 \cos ( \alpha )}{\pi w^2} E_{\text {p}} , \end{aligned}$$

where $E_{\text {p}}$ denotes the pulse energy. At $\alpha ={89}^{\circ }$, and a wavelength of 1 μm, the reflectivity of an uncoated quartz surface for s-polarized light is 94%. By avoiding a mirror with a coating, which contain high-refractive-index material and typically come with small deposition errors and contaminants, the highest possible damage threshold can be achieved. The output coupler, being a quartz (or sapphire, diamond, etc.) plate, can be readily and cost-effectively replaced. For a given switching time and rate, a larger spot size can be accommodated by increasing the overall size of the system. Thus, the maximum peak power scales with the square of the system size (Eqs. 4, 5).

## Planar double-mirror geometry

**Fig. 3:**
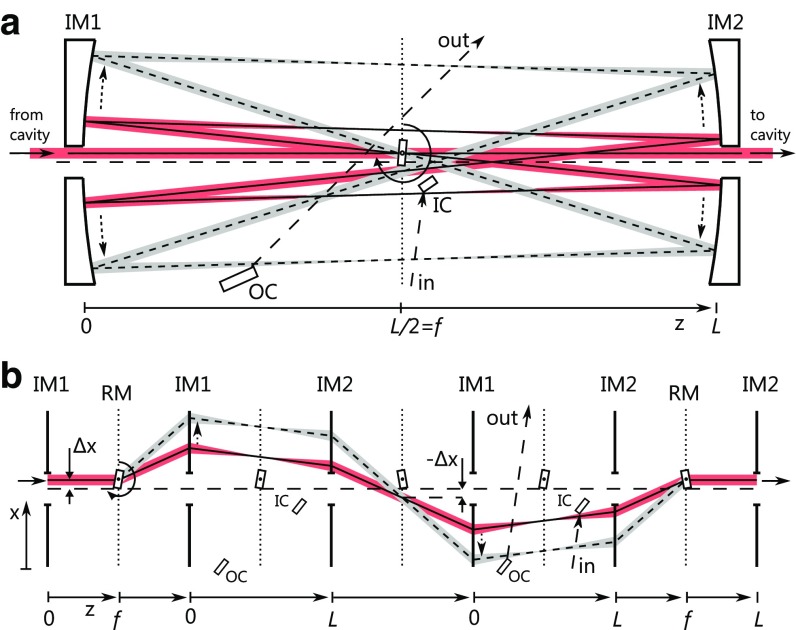
**a** Top view of the planar two-mirror geometry with a two-faceted rotor (RM), two imaging mirrors (IM1, IM2), an input coupling mirror (IC), and an output coupling mirror (OC). The beam path is shown for two rotation angles (*solid line* just after input coupling, *dashed line* just before output coupling). **b** Schematic view of the beam path. The beam passes each of the imaging mirrors twice

In principle, the pulse-picker geometries discussed so far allow for an unobstructed deflection beam path for all rotation angles (apart from input or output couplers). Thus, they can provide a resonator with an open beam path for most of the rotation period, which corresponds to the input and/or output switching rate. However, in regenerative amplifiers for instance, the time between input coupling to and output coupling from the cavity is often only a fraction of the repetition period. Thus, the cavity needs to be closed for just a small range of rotation angles of the rotating mirrors. In the case of the two pulse-picker variants discussed so far, smaller imaging mirrors covering only the necessary rotation angles could be used. In addition, applications of this kind would allow for the use of a particularly advantageous third geometry, as shown in Fig. 3a. In contrast to the previous implementation, the axis of rotation of the rotating mirrors is perpendicular to the incoming beam path. The deflection beam paths for different rotation angles form a plane. The mirror surfaces of the rotor are parallel to the rotation axis and to each other. The imaging mirror setup is similar to the second variant, with the focal planes coinciding with the rotating mirror surfaces. However, the central axis along which the imaging mirrors are aligned is offset from the incoming beam and the rotating mirrors by a distance $\Delta x$. Figure 3b illustrates the beam path in the system. The 4-f configuration of the imaging mirrors creates an inverted image in the central plane, after the beam has passed each of the imaging mirrors once. In this plane, the beam is offset by $-\Delta x$ from the central axis. If $\Delta x$ is larger than the maximum radius of the rotor, the beam passes the rotor without being clipped and enters the imaging setup for a second time. Two passes through the 4-f imaging setup create an upright image of the first rotating mirror surface on the parallel second surface. The reflection from this surface produces a stationary output beam from the rotation-angle-dependent deflection beams for a continuous range of rotation angles. As in the second variant, the (close to) collimated input beam is focused in the Fourier planes in the symmetry plane, and input or output coupling elements can be placed at some position of the deflection beam having a suitable beam size. A major advantage of this geometry is its inherent insensitivity to vibrations and other deviations from a perfect rotation of the rotor: Any kind of angular or positional error of the first surface is reproduced by the second surface and, due to the imaging system, its effects on the output beam cancel out.

The rotor can be a two-faced substrate, or have a geometry with a larger number of parallel surface pairs, i.e., square, hexagonal, and so forth. A simple two-faced rotor would allow for a switching rate corresponding to twice the rotation frequency. Rotors with a higher number of facets would further increase the maximum switching rate. For small deflection angles, the transverse separation of the paths of subsequent pulses is

6 $$\begin{aligned} \Delta s_3 = 2 \pi L \frac{f_{\text {rot}}}{f_{\text {rep}}}. \end{aligned}$$

In contrast to the first two variants, this separation does not depend on the deflection angle. Consequently, a much larger separation or, alternatively, a much shorter switching time can be achieved in a system with the same footprint. The deflection angle $\varTheta$ equals twice the rotation angle, i.e., the angle of incidence on the rotating mirrors. The maximum range of deflection angles $\Delta \varTheta$ for which the cavity is open is given by the minimum angle $\varTheta _\text {min}$ for which the beam is not clipped by the rotating mirror in the first Fourier plane (Fig. 3b), and a maximum angle $\varTheta _\text {max}$ given by the dimensions of the imaging mirrors. The maximum number of round-trips *N* which a pulse can undergo in a cavity containing this pulse picker is

7 $$\begin{aligned} N_\text {plan}= \frac{\varTheta _\text {max} - \varTheta _\text {min}}{4 \pi } \frac{f_{\text {rep}}}{f_{\text {rot}}}. \end{aligned}$$

Since the angle of incidence on the rotating mirrors is not constant, rotation-angle-dependent astigmatism will occur when spherical imaging mirrors are used. These changes in the effective focal length of the imaging mirrors with the rotation angle will cause a varying ellipticity of the cavity mode. If large deflection angles are required, the use of parabolic imaging mirrors might be necessary (also depending on the sensitivity of the surrounding cavity). In this geometry, the rotation of the mirrors during the propagation time in the device causes a small tilt of the output beam that is constant for a given rotation frequency.

## Nonplanar double-mirror geometry

**Fig. 4:**
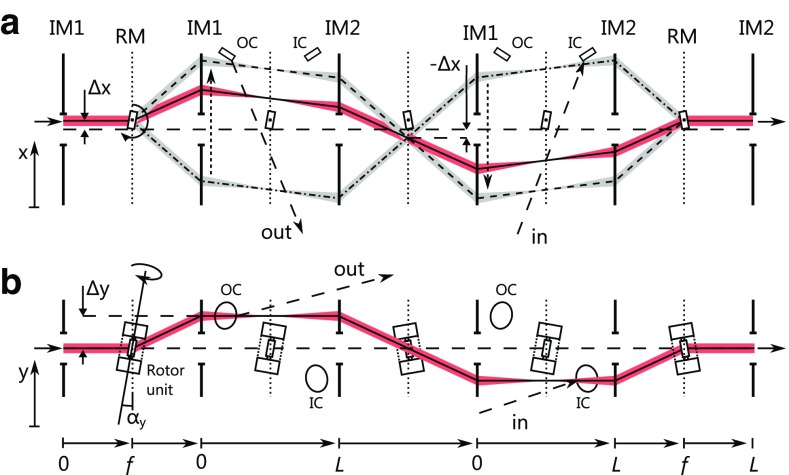
**a** Schematic top view of the beam path in the nonplanar two-mirror geometry for three rotation angles (*dash-dotted line* path of a pulse just after input coupling, *dashed line* just before output coupling, *solid line* in between). **b** Schematic side view of the beam path. The inclined rotation axis allows the beam path to circumvent the rotor unit and the opening in the imaging mirrors

Figure 4 shows a simple modification of the planar double-mirror geometry with which the maximum deflection angle to achieve a specific number of round-trips can be reduced by more than a factor of two. Here, the rotating mirror and its rotation axis are inclined by an angle $\alpha _\text {y}$ in the $z{-}y$ plane with respect to the symmetry plane of the imaging setup, offsetting the beam by $\Delta y$ in the first Fourier plane, and $-\Delta y$ in the second Fourier plane (Fig. 4b). With this modification, the deflection beam circumvents the rotor and its bearing and the holes in the imaging mirrors, even for zero deflection angle in the $x-z$ plane. Thus, there is an unobstructed path for the deflection beam for all deflection angles between $-\varTheta _\text {max}$ and $\varTheta _\text {max}$, and $\Delta \varTheta$ is equal to $2 \varTheta _\text {max}$. The maximum number of round-trips is now

8 $$\begin{aligned} N_\text {nonplan}= \frac{\varTheta _\text {max}}{2 \pi } \frac{f_{\text {rep}}}{f_{\text {rot}}} . \end{aligned}$$

The minimum width *X* of the imaging mirrors is

9 $$\begin{aligned} X_\text {nonplan}= N \Delta s_3 . \end{aligned}$$

## Application example 1: regenerative amplifier

The nonplanar double-mirror geometry would be particularly suited for regenerative amplifiers. With rotation frequencies of several kHz and multifaceted rotors, switching rates of up to a few 10 kHz should be achievable. We exemplify practical pulse-picker parameters based on an amplifier build by Nubbemeyer et al. [20], who have recently achieved an average power of about 1 kW at an output repetition rate of either 5 or 10 kHz, with 40 round-trips and a cavity repetition rate of 20 MHz. A nonplanar double-mirror optomechanical switch with a two-surface rotor at a rotation frequency of 5 kHz would allow an output repetition rate of both 5 or 10 kHz. A magnetically beared motor which could be suitable for this application is commercially available [21]. Its length along the rotation axis is 85 mm. To achieve 40 round-trips, $\Delta \varTheta$ has to be ${7.2}^{\circ }$ (Eq. 8), resulting in a maximum angle of incidence of ${1.8}^{\circ }$ in the $z{-}x$ plane. With a distance of 1 m between the imaging mirrors, a pulse path separation of 1.57 mm would be achieved (Eq. 6). The dimensions of the motor would imply a $\Delta y$ of about 45 mm, and thus an angle of incidence of ${3}^{\circ }$ in the $z{-}y$ plane. The input/output coupling plate could be placed at a position where the beam radius is 0.7 mm. With an angle of incidence of ${88}^{\circ }$, the spot on the plate would be elongated to a *w* of 20 mm. For a Gaussian profile with this spot size and a pulse energy of 200 mJ, the peak fluence is about ${0.9}\hbox { J cm}^{-2}$ (Eq. 5). At a central wavelength of 1053 nm and a pulse duration of 1 ns, the damage fluence of fused silica is about $40\hbox { J cm}^{-2}$; for a pulse duration of 1 ps, it is $2\hbox { J cm}^{-2}$ [22]. Shorter amplifier cavities would allow for a larger number of round-trips, but increase the fluence on the output coupler in a system with identical dimensions. Higher output rates can be achieved by using multifaceted rotors. In conclusion, the outlined optomechanical pulse picker could tolerate higher pulse energies, and/or far shorter pulses than state-of-the-art Pockels cells. Apart from the higher damage threshold, the losses, nonlinearity, thermal lensing, and dispersion associated with the transmission through the Pockels cell would be avoided. An optomechanical pulse picker would likely require vacuum for stable operation.

## Application example 2: Stack-and-dump cavity

In Ref. [7], a stack-and-dump cavity with a repetition rate of 10 MHz is proposed as a part of a future fiber-laser-based particle accelerator. From this cavity, an output pulse energy of 1.2 J with pulses stretched to 4 ns is envisaged. The pulse train seeding the cavity is continuous, making a pulse picker that provides an unobstructed beam path for most of the output repetition period necessary. We outline practical parameters for an optomechanical pulse picker of a circular double-mirror geometry as discussed in Sect. 4, and a rotation frequency of 8.3 kHz [15]. With this output switching rate, the maximum number of stacked pulses is 1200. With a length of 1.5 m, a distance between the rotating mirrors of 70 mm and an angle of incidence of ${2}^{\circ }$, the diameter of the deflection beam circle on the imaging mirrors is about 100 mm. At an angle of incidence of ${2}^{\circ }$, birefringent effects in typical broadband highly reflective mirrors are negligible even for the largest bandwidths yet demonstrated in high-finesse ECs [23]. The separation of the optical axes of successive pulses is 0.26 mm (Eq. 4). For a pulse energy of 1.2 J and a beam radius of 130 μm at an output coupler with ${89}^{\circ }$ angle of incidence, the peak fluence would be $79\hbox { J cm}^{-2}$. Using the $\tau ^{0.5}$-law for the pulse width dependence of the damage threshold, a damage threshold of fused silica of about $80\hbox { J cm}^{-2}$ for a pulse duration of 4 ns can be extrapolated from the measurements in [22], putting the highly ambitious pulse energy targeted in [7] within reach.

## Misalignment sensitivity

**Fig. 5:**
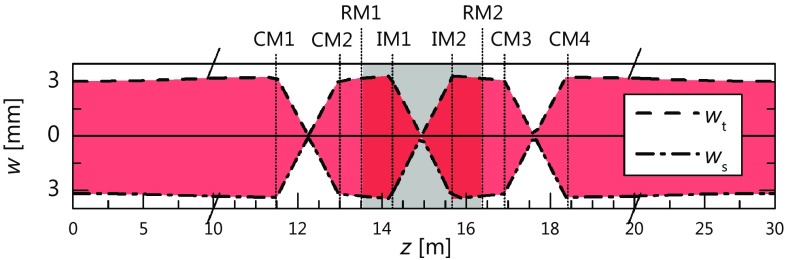
Calculated sagittal and tangential $1/e^2$-intensity radius ($w_\text {s}, w_\text {t}$) for the 10-MHz EC outlined in Sect. 8 containing a pulse picker of the circular two-mirror geometry (RM1, IM1, IM2, RM2), and four additional curved mirrors (CM1-4)

The feasibility of the concept critically depends on the perturbations of the beam caused by the rotation of the mirrors, and on the sensitivity of the application to these. While the imaging configuration ideally results in a stationary output beam, misalignments, manufacturing tolerances, and astigmatism in spherical imaging mirrors can cause a rotation-angle-dependent translation and ellipticity of the mode. An important question for intra-resonator applications is whether the resulting periodic change in the beam path and shape causes any significant effects that would not be present for a static misalignment or astigmatism. If so, such effects would be particularly severe for enhancement cavities, where the circulating pulse train needs to overlap spatially, temporally, and in terms of its polarization with an incoming pulse train. To address this question, we simulate the enhancement in a 10-MHz EC including an optomechanical pulse picker as outlined in the previous section, giving a maximum number of 1200 round-trips between two switching events. To be able to tune the position in the cavity’s stability range, the cavity should include additional concave mirrors. In a cavity with a round-trip Gouy phase close to an odd multiple of $\pi$, large spots on the mirrors can be achieved while avoiding a high misalignment sensitivity [19]. In our cavity example, two pairs of concave mirrors with a radius of curvature of 1500 mm are used. With a distance of 1470 mm between each of the two curved mirror pairs and a wavelength of 1040 nm, the beam radius is about 3 mm on all cavity mirrors (Fig. 5). Assuming the same footprint as in [14], 20 flat folding mirrors producing overall losses of about 400 ppm are needed. To achieve a good stacking efficiency with 1150 round-trips, an input coupler of 99.75% reflectivity is chosen.

Because of the rotating deflection beam path, the cavity cannot be described using “global” sagittal and tangential planes. Here, we simulate the buildup in the cavity numerically in 3D using a modified Fox-Li algorithm [24]. The beam size and divergence of a round input beam is optimized for the cavity with an inactive pulse picker (rotating mirrors are at rest). In this case, the simulation yields an enhancement of about 780 after 1150 round-trips, corresponding to a stacking efficiency of 0.65. The angle of incidence of ${2}^{\circ }$ on the imaging mirrors causes an eccentricity of the beam of 0.27 at the curved cavity mirrors. The simulation for an active pulse picker shows a periodic rotation of the major axis of the elliptic intracavity mode profile with the deflection beam path, but no change in the enhancement level. When a tilt of one of the rotating mirrors is introduced, the central axis of the cavity beam is misaligned. For the active pulse picker, the misaligned beam axis periodically rotates around the axis of the unperturbed beam. The enhancement level drops by the same amount for both the active and inactive pulse picker. The loss of enhancement stems exclusively from the decrease in the spatial overlap of the intracavity beam with the seeding beam. Thus, we can follow the approach taken in [19], which uses far less computation time, to investigate the misalignment sensitivity of the system in detail.

We find that in the cavity example used above, positioning errors or tilts of the imaging mirrors also lead to a rotating cavity beam axis. For instance, a quite large positioning error of one of the imaging mirrors of 5 mm along the *z*-axis results in a loss of overlap with a fixed input beam of about 10%. A tilt of one of the imaging mirrors by 10 μrad results in an overlap reduction of 4%. A tilt of the rotation axis and the rotor with respect to the imaging mirrors causes a stationary misalignment, but no rotation of the cavity beam axis. For pulse pickers of the circular two-mirror geometry, the rotating mirror surfaces should have identical inclination angles and orientations with respect to the axis of rotation. Errors in the inclination angle can be compensated by adjusting the distances of the rotating to the stationary mirrors to change the magnification of the imaging system. For example, for an error of the inclination angle of 100 μrad, a nearly static cavity beam can be achieved by repositioning both imaging mirrors by about 20 mm along the *z*-axis. However, the imaging setup cannot compensate for different orientations of the mirrors, i.e., when the planes of the inclination angles of the mirrors are not identical. The effective mirror tilt resulting from this effect is described by Eq. 3, with $\Delta \phi$ now representing the orientation mismatch. In the cavity simulation, an orientation mismatch of 500 μrad results in a rotating offset of the intracavity mode axis of about 0.2 mm at the curved cavity mirrors and the overlap decreases by 10%. Since the rotor has to meet high mechanical demands, and its mirrors will most probably not be adjustable, achieving the necessary accuracy in the orientation of the surfaces will be critical. Importantly, the sensitivity of the EC with respect to misalignments of the mirrors comprising the pulse picker is not higher than for the other cavity mirrors. Furthermore, all perturbations are periodic and synchronized to the output repetition rate, resulting in a stable output. While a detailed analysis of all possible perturbations exceeds the scope of the paper, we conclude that we see no fundamental limitations related to the alignment sensitivity of the optomechanical pulse picker.

## Sensitivity to rotor motion irregularities

Another important aspect of the technical implementation of this concept will be deviations of the actual rotor motion from a perfect rotation caused by, e.g., unbalance. These can be classified in three categories: parallel deviations (shifts) and angular deviations (tilts) of the rotation axis, and axial displacements of the rotor along the rotation axis. The planar and nonplanar double-mirror geometries are inherently insensitive to these effects. Here, the upright imaging of the front surface of the compact rotor onto its back surface results in a self-compensation of positional and angular errors of the rotor position. Axial displacements are parallel to the rotor surfaces. For the circular one-mirror geometry, in contrast, axial movements would directly translate to changes in the cavity length and, therefore, render its use for applications requiring interferometric stability challenging. Parallel rotation errors would, to a much lesser extent, also affect the cavity length. The imaging configuration results in self-compensation of angular rotation errors. The circular two-mirror geometry is insensitive to length changes due to axial rotor positioning errors, since the displacement of the first rotating mirror surface is compensated by an identical displacement of the second surface. However, due to the inverted imaging configuration in this geometry, angular errors of the rotor position result in a displacement of the cavity beam path. For the stack-and-dump-cavity example described in the previous section, an angular error of 10 μrad corresponding to a rotor tip displacement of 0.35 μm would result in a 14% decrease in overlap. In [15], a maximum displacement of the rotor tip of 17 μm is measured at a rotation frequency of 8.7 kHz. These measurements were taken with an unbalanced rotor, and it is not stated how much of the displacement is due to angular deviations. Still, the results suggest that the balancing of the rotor and the optimization of its active stabilization may be the main technological challenges for an application of the concept in enhancement cavities. For the regenerative amplifier application discussed in Sect. 6, the effects of the rotor displacements reported in [15] would be negligible.

## Conclusion

In this article, we have outlined a concept for a family of ultrafast all-reflective pulse pickers. Discussing three specific geometries, we have shown that such optomechanical pulse pickers could particularly benefit high-power and high-pulse energy applications. Designs suitable for regenerative amplifiers could support pulse energies of several joules and repetition rates of up to a few tens of kHz, while circumventing the losses, dispersion, and the nonlinear and thermal effects in state-of-the-art Pockels cells. Optomechanical pulse picking could additionally render the temporal stretching of pulses in such systems unnecessary. The concept also holds promise for cavity dumping in passive enhancement cavities. Here, output repetition rates of up to several kHz could potentially be reached for joule-level output pulse energies. Other applications may emerge in wavelength ranges where conventional pulse pickers are not available, in particular in the terahertz [25] and X-ray [26] ranges.
